# Performance of the new Laryngeal Tube LT^®^evo during simulated resuscitation: a randomised crossover manikin study

**DOI:** 10.1186/s12245-026-01163-8

**Published:** 2026-03-24

**Authors:** Mathini Vaseekaran, Harald Genzwürker, Jochen Hinkelbein, Annika Hoyer, Julia Johanna Grannemann, Lydia Johnson Kolaparambil Varghese, Tobias Vollmer, Gerrit Jansen

**Affiliations:** 1 Ruhr University Bochum, Johannes Wesling Hospital Minden, University Department of Anaesthesiology, Intensive Care Medicine, Emergency Medicine and Pain Management, Minden, Germany; 2 Medical Faculty, University of Heidelberg, Heidelberg, Germany; 3 Bielefeld University, Medical School OWL, Biostatistics and Medical Biometry, Bielefeld, Germany; 4 Emergency Medical Service District of Guetersloh, Guetersloh, Germany

**Keywords:** Airway management, Cardiopulmonary resuscitation, Supraglottic airway device, Simulation study, Emergency medicine, Laryngeal tube

## Abstract

**Background:**

Effective airway management is essential for high-quality cardiopulmonary resuscitation (CPR). While tracheal intubation (TI) has traditionally been regarded as the reference technique for advanced airway management, it requires substantial expertise and may prolong no-flow time during CPR. Current European Resuscitation Council (ERC) guidelines recommend the i-gel^®^ as the preferred supraglottic airway (SGA). The newly developed Laryngeal Tube LT^®^evo (VBM Medizintechnik, Germany) was designed to simplify insertion and improve ventilation performance. This study evaluated its efficacy compared with bag-valve-mask-ventilation (BVM) and tracheal intubation (TI) in a standardized resuscitation scenario.

**Methods:**

In this prospective, randomised crossover manikin study, 20 anaesthesiologists with varying experience in prehospital and in-hospital Airway management during CPR performed three resuscitation scenarios using BVM, TI, or LT^®^evo in random order. Each 5-minute scenario simulated a cardiac arrest with initial shockable rhythm and required up to three mandatory defibrillations. The primary endpoint was total no-flow time. Secondary endpoints included device insertion time, time-to-first-effective-ventilation (tidal-volume ≥ 400 mL), and number of insufficient ventilations.

**Results:**

All 60 simulations were completed. Compared with LT^®^evo, BVM produced statistically longer no-flow time (regression coefficient [RC] = 4.00; 95%-CI = 0.13–7.87), a higher rate of insufficient ventilations (RC = 1.25; 95%CI = 0.44–2.09), and lower tidal-volumes (RC = − 66.05; 95%CI = − 124.75- −7.35). Compared to LT^®^evo, there was no evidence for a difference in no-flow time for TI (RC = − 2.17; 95%CI = − 6.04–1.70) in experienced users.

**Conclusion:**

In this first evaluation of the LT^®^evo, the device enabled rapid, effective ventilation with minimal interruption of chest compressions. Its ease of use and observed performance characteristics support further clinical investigation as potential alternative SGA for resuscitation.

## Introduction

Effective airway management is a cornerstone of high-quality cardiopulmonary resuscitation (CPR), directly influencing oxygenation, ventilation, and ultimately patient outcomes [1–3]. While tracheal intubation (TI) has traditionally been regarded as the reference technique for advanced airway management, it requires significant training and may lead to interruptions in chest compressions - increasing no-flow time - which are associated with poorer survival and neurological outcomes [3–5].

The newly released European Resuscitation Council (ERC) Guidelines 2025 emphasize a provider-competence-based approach, recommending TI only for rescuers with a high likelihood of first-pass success [4]. For many emergency providers, supraglottic airways (SGA) represent a safer and faster alternative that minimises CPR interruptions [6]. SGAs have become an integral part of advanced life support algorithms [1, 7, 8] and are associated with similar survival outcomes compared with TI in both prehospital and in-hospital cardiac arrest environments [4].

**Fig. 1:**
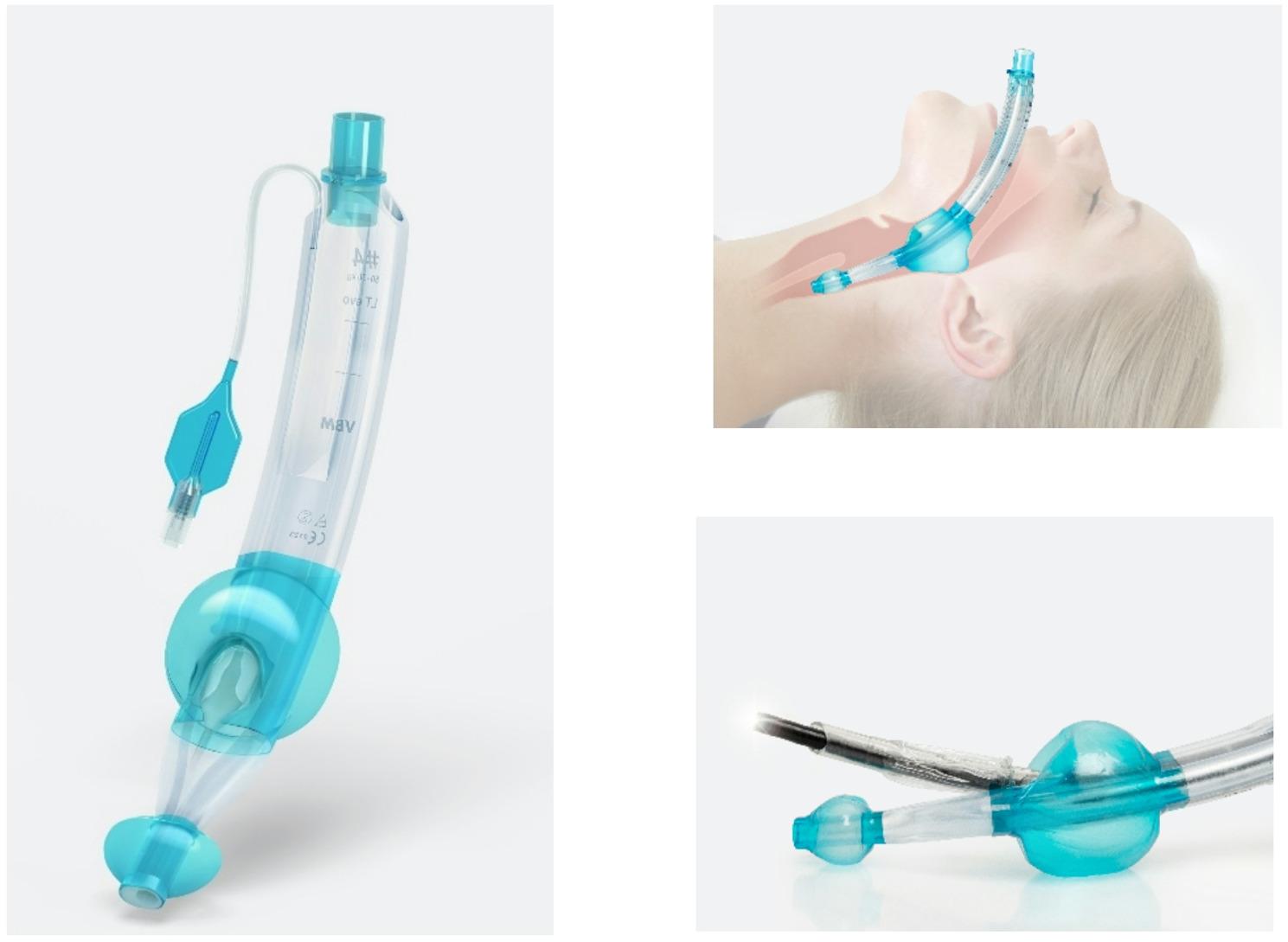
Laryngeal Tube LT^®^evo (Image source: VBM Medizintechnik GmbH)

In the last decades, laryngeal tubes such as the LTS-D have been widely used in prehospital and in-hospital resuscitation; however, current ERC guidelines specify i-gel^®^ as the first-line SGA device for airway management during CPR [4]. This shift reflects evidence supporting i-gel^®^’s ease of insertion, time advantage due to a non-inflatable cuff, and reliable seal pressures. Yet, laryngeal tubes have evolved significantly, and the feasibility and workflow characteristics of the new laryngeal tube LT^®^evo (VBM Medizintechnik GmbH, Germany) can now be assessed as a first step in exploring its potential role in airway management. LT^®^ evo is a newly developed laryngeal tube belonging to the group of second-generation SGA as a redesigned version of the established LTS-D. It features a redesigned tip to support correct insertion, a widened ventilation lumen allowing subsequent fibreoptic-guided intubation, optimized cuff geometry to enhance seal with low pressure, and an additional channel allowing insertion of a gastric tube (See Fig. 1). The LT^®^evo incorporates design elements of the LTS-D together with structural features of laryngeal mask-type supraglottic airways, with the intention of combining functional aspects of both designs. These modifications aim to facilitate sufficient ventilation and simplify use under resuscitation conditions.

To date, no data exist on the performance of the LT^®^evo during CPR. This study therefore sought to evaluate its performance in comparison with BVM and TI in a standardized simulated Out-of-Hospital Cardiac Arrest (OHCA) scenario.

We hypothesized that the LT^®^evo would allow rapid establishment of an advanced airway with acceptable ventilation parameters and process-related outcomes, including no-flow time, within the applied resuscitation algorithm.

As a study in a simulated setting, the present investigation does not aim to compare supraglottic airway devices, but to assess feasibility and workflow characteristics of the LT^®^evo during CPR.

## Materials and methods

### Study design and ethical approval

This prospective, randomised, crossover manikin study was conducted between 20th of May and 10th of June 2025 at the simulation centre of the University Hospital Minden, Germany. Ethical approval was obtained from the Ethics Committee of the Ruhr-University Bochum, Campus East Westphalia (Reference: 2025 − 1362, Approval Date: 26.05.2025). All participants provided written informed consent prior to enrolment.

### Sample size calculation

Sample size calculation was conducted prior to start of the study for the primary outcome of no-flow time. This calculation was based on findings reported by Wiese et al. for the comparison of LTS-D, BVM and TI [9]. The authors reported a no-flow time of 125 (12.48), 160 (12.04) and 207 (37.37) for LTS-D, BMV and TI, respectively. Based on these findings, t-tests for each comparison with a Bonferroni-correction for multiple testing and a power of 80%, we determined a sample size of 20 study participants.

### Participants

20 anaesthesiologists with both in-hospital and prehospital emergency medicine experience were recruited. All had undergone Advanced-Cardiac-Life-Support (ACLS) training at the University Hospital Minden and were actively involved in emergency medical response teams. Participant characteristics are summarised in Table 1.

**Table 1:** Participant characteristics

Variable	Mean ± SD / *n* (%)
Age (years)	34.4 ± 6.5
Experience as physician (years)	6.8 ± 5.5
Experience as EMS physician (years)	3.7 ± 5.2
Number of airway procedures/year	655.0 ± 297.8
Sex: male	9 (45%)
Sex: female	11 (55%)

Baseline demographic and professional characteristics of the participating anaesthesiologists. Data are presented as mean mean ± standard deviation or n (%). Abbreviations: EMS, Emergency Medical Service; SD, standard deviation

### Study protocol

Each participant performed three standardised resuscitation scenarios using BVM, TI, and the LT^®^evo. At the time of the study, the LT^®^evo was not yet CE-marked. Nevertheless, it represented a fully functional manufacturer-provided device (non-prototype) and was used exclusively for simulation purposes without any clinical application. The sequence of device use was randomized using block randomization with a block length of three. Prior to the first use of the LT^®^evo, all participants received a brief standardised instruction from the same experienced instructor and were given the chance to try the handling of the new device in the manikin.

Each scenario simulated an OHCA with an initial shockable rhythm and a total duration of five minutes. Simulations were conducted in teams of two: one participant acted as team leader and performed airway management, while the second performed chest compressions and defibrillation. Teams were formed consecutively according to order of inclusion. The sequence of airway devices was randomized prior to study initiation using a computer-generated randomization list. Each participant performed the three airway strategies in a predefined randomized order. Roles were switched between scenarios.

All simulations followed a standardized 30:2 compression-to-ventilation ratio, including after placement of TI or the LT^®^evo, to ensure comparability across groups. Prior to insertion of an advanced airway (TI or LT^®^evo), at least two effective BVM ventilations were required.

Advanced airway placement was permitted after the initial rhythm analysis and first defibrillation attempt in accordance with the ACLS algorithm. The simulation began with a shockable rhythm, and defibrillation was allowed as soon as technically feasible without imposed delay.

To minimize rescuer fatigue, chest compression providers were exchanged, as per guideline recommendation, approximately every two minutes. When required for the compressor to change, brief assistance by an additional person was permitted to allow continuous workflow; however, airway management and study-related interventions were performed exclusively by the designated participant.

### Simulation equipment

Simulations were performed using the Resusci Anne Advanced Skilltrainer (Laerdal™ Inc., Stavanger, Norway), connected to the Laerdal™ PC Skill Reporting Software for automatic recording of ventilation and timing metrics. All airway devices were used according to respective manufacturer recommendations.

### Outcomes

The following parameters were recorded: time-to-first-effective-ventilation (sec); defined as a tidal volume > 400 ml; device insertion time (sec), defined as the interval from picking up the device to successful cuff inflation ; first-past-success; achieved tidal volume (ml); number of insufficient ventilations (tidal volume < 400 mL); no-flow-time (sec), defined as the cumulative period without chest compressions including pauses for ventilation according to the applied 30:2 resuscitation algorithm; time-to-first-defibrillation (sec) and the timing and completion of up to three defibrillation shocks according to ACLS algorithm. Time-to-first-effective-ventilation was measured from scenario start and included the initial two mandatory bag-valve-mask ventilations, reflecting the realistic delay in availability of an advanced airway device. After each scenario, participants completed a standardized questionnaire assessing handling, perceived safety, and overall performance of each technique using a 5-point-likert-scale (1 = very poor; 5 = very good).

The primary outcome was total no-flow time. Secondary outcomes included time-to-first-effective-ventilation, device insertion time, tidal volumes achieved, number of insufficient ventilations and time to first defibrillation.

### Statistics

Data were first analysed descriptively according to the scale level of the variables: mean and standard deviation were reported for continuous variables and numbers and proportions for categorical variables. For the primary outcome total no-flow time, a linear mixed regression model with random intercept was used to account for potential intra-participant correlations. For continuous secondary outcomes, again linear mixed regression models with random intercepts were applied. For binary outcomes, a logistic mixed regression model with random intercept was used. As effect measures, we report on mean differences or odds ratios with 95% confidence intervals and p-values if appropriate. A p-value ≤ 0.05 was considered statistically significant. All analyses were conducted using the statistical software SAS 9.4 (SAS Institute Inc., Cary, NC).

## Results

### Participant characteristics

A total of 20 anaesthesiologists participated in the study (11 female, 9 male; mean age 34.4 ± 6.5 years). The average professional experience was 6.8 ± 5.5 years, with 3.7 ± 5.2 years of emergency medical service (EMS) experience. Participants reported performing a mean of 655 ± 298 airway procedures annually (Table 1).

### Airway performance and primary outcome

All 60 simulated resuscitation scenarios were completed successfully. No-flow time differed between airway management strategies within the standardized 30:2 resuscitation algorithm. Compared to the LT^®^evo, BVM was associated with a longer no-flow time (regression coefficient [RC] = 4.00, 95%CI = 0.13–7.87; *p* = 0.0433). TI showed no statistically significant difference in no-flow time relative to LT^®^evo (RC = − 2.17; 95%CI = − 6.04–1.70; *p* = 0.2642). Overall mean no-flow times were 67.7 ± 13.8 s for BVM, 61.5 ± 10.9 s for TI, and 63.7 ± 9.2 s for LT^®^evo (Table 2).

**Table 2:** Descriptive results by airway device (mean ± standard deviation)

Variable	Total (*n* = 60)	BVM (*n* = 20)	TI (*n* = 20)	LT^®^evo (*n* = 20)
No-flow time (s)	64.3 ± 11.6	67.7 ± 13.8	61.5 ± 10.9	63.7 ± 9.2
Time to first effective ventilation (s)	75.1 ± 22.7	56.5 ± 17.1	95.1 ± 17.2	73.8 ± 14.8
Device insertion time (s)	8.1 ± 10.2	0.0 ± 0.0	17.9 ± 11.5	6.5 ± 4.1
Number of insufficient ventilations	0.9 ± 1.4	1.6 ± 1.9	0.8 ± 1.1	0.4 ± 0.7
Tidal volume (mL)	650.2 ± 125.5	611.0 ± 135.2	662.5 ± 132.0	677.0 ± 103.2
Time to first defibrillation (s)	52.3 ± 14.8	52.0 ± 16.3	53.2 ± 14.9	51.8 ± 13.9
3 Defibrillations completed in 300s – reached	51 (85.0%)	16 (80.0%)	17 (85.0%)	18 (90.0%)

Primary and secondary outcome measures for all simulations and stratified by airway device (BVM, TI, LT^®^evo). Outcomes include no-flow time, ventilation parameters, insertion characteristics, and defibrillation metrics. Data are presented as mean (SD) or number (%). Effective ventilation was defined as tidal volume ≥ 400 mL. Abbreviations: BVM, bag-valve-mask ventilation; TI, tracheal intubation; SD, standard deviation

### Secondary outcomes

The LT^®^evo enabled faster airway establishment when compared to TI. Device insertion time was statistically shorter with LT^®^evo compared with TI (RC = 11.45; 95%CI = 7.45–15.45; *p* < 0.0001), and time-to-first-effective-ventilation (tidal volume ≥ 400 mL) was also faster (RC = 21.25; 95%CI = 12.86–29.64; *p* < 0.0001) (Table 4).

In contrast, BVM achieved earlier first ventilations (RC = − 17.4; 95%CI − 25.8- − 9.0; *p* < 0.001), but was associated with inferior ventilation quality and longer overall interruptions in chest compressions (Table 3).

**Table 3:** Results of the linear regression analysis with random intercept comparing BVM vs. LT^®^evo

Outcome	Mean difference between BVM and LT^®^evo	95% CI	*p*-value
No-flow time (s)	4.00	[0.13; 7.87]	0.0433
Time to first effective ventilation (s)	−17.40	[− 25.79;−9.01]	0.0002
Insufficient ventilations	1.25	[0.44; 2.09]	0.0047
Tidal volume (ml)	−66.05	[− 124.75; −7.35]	0.0284

Results of linear mixed model analyses comparing bag-valve-mask ventilation with LT^®^evo for selected process and ventilation outcomes. Effect estimates are presented as mean differences with 95% confidence intervals. Abbreviations: BVM, bag-valve-mask ventilation; CI, confidence interval

The number of insufficient ventilations (tidal volume < 400 mL) was highest with BVM (1.6 ± 1.9), followed by TI (0.8 ± 1.1), and lowest with LT^®^evo. Mean tidal volumes achieved were 611 ± 135 mL for BVM, 663 ± 132 mL for TI, and 677 ± 103 mL for LT^®^evo. Descriptive statistics for all devices are shown in Table 2, and regression analyses in Tables 3 and 4.

**Table 4:** Results of the linear regression analysis with random intercept comparing TI vs. LT^®^evo

Outcome	Mean difference between TI and LT^®^evo	95% CI	*p*-value
No-flow time (s)	-2.17	[-6.04; 1.70]	0.2642
Device insertion time (s)	11.45	[7.45–15.45]	< 0.0001
Time to first effective ventilation (s)	21.25	[12.86–29.64]	< 0.0001
Insufficient ventilations	0.40	[-0.44; 1.24]	0.3421
Tidal volume (ml)	-14.50	[-73.20; 44.20]	0.6199

Results of linear mixed model analyses comparing tracheal intubation with LT^®^evo for selected process and ventilation outcomes. Effect estimates are presented as mean differences with 95% confidence intervals. Abbreviations: TI, tracheal intubation; CI, confidence interval

Within the 5 min scenario, three defibrillations were completed by 90% of participants using the LT^®^evo, 85% using TI, and 80% using BVM.

### Subjective evaluation

After each simulation, participants completed a standardized questionnaire assessing each airway technique in terms of handling, placement stability, perceived safety, placement effort, and overall impression using a 5-point Likert scale.

The LT^®^evo and TI achieved identical median overall ratings of 4.0. The interquartile range was wider for LT^®^evo (Q1; Q3: 4.0; 5.0) compared to TI (4.0; 4.0), while BVM showed a lower median rating of 3.0 (3.0; 4.5). In terms of handling, the LT^®^evo achieved a median of 5.0 (5.0; 5.0), while TI and BVM both reached 4.5 (TI: 4.5 [4.0; 5.0]; BVM: 4.5 [3.5; 5.0]). For placement stability, both the LT^®^evo and TI were rated equally at 5.0 (4.0; 5.0), compared to BVM with 3.0 (2.0; 5.0). The perceived effort of placement was lowest (i.e., easiest) for the LT^®^evo and BVM, each with 5.0 (5.0; 5.0), while TI was rated considerably lower at 3.0 (1.5; 4.5). Regarding perceived safety, TI was rated highest with 5.0 (4.5; 5.0), followed by LT^®^evo with 4.0 (4.0; 5.0) and BVM with 3.0 (1.5; 4.5). Table 5 summarizes the distribution of all subjective ratings across the five evaluated dimensions (handling, placement effort, perceived safety, placement stability, and overall impression) for each airway management technique.

**Table 5:** Distribution of Subjective Ratings (5-Point Likert Scale)

Variable	Total (*n* = 60) Median (Q1–Q3)	BVM (*n* = 20) Median (Q1–Q3)	TI (*n* = 20) Median (Q1–Q3)	LT^®^evo (*n* = 20) Median (Q1–Q3)
Handling	5.0 (4.0–5.0)	4.5 (3.5–5.0)	4.5 (4.0–5.0)	5.0 (5.0–5.0)
Placement effort	5.0 (3.0–5.0)	5.0 (4.5–5.0)	3.0 (1.5–4.5)	5.0 (5.0–5.0)
Perceived safety	4.0 (3.0–5.0)	3.0 (1.5–4.5)	5.0 (4.5–5.0)	4.0 (4.0–5.0)
Placement stability	5.0 (3.0–5.0)	3.0 (2.0–5.0)	5.0 (4.0–5.0)	5.0 (4.0–5.0)
Overall impression	4.0 (3.0–5.0)	3.0 (3.0–4.5)	4.0 (4.0–4.0)	4.0 (4.0–5.0)

Subjective evaluation of airway management techniques assessed using a 5-point Likert scale (1 = very poor; 5 = very good). Data are presented as median and interquartile range (Q1–Q3)

## Discussion

This study investigated the performance of the newly developed LT^®^evo in a simulated OHCA scenario and compared it with BVM and TI. The main findings in this setting are threefold: (i) LT^®^evo enabled rapid airway establishment and early effective ventilation with minimal interruption of chest compressions. (ii) Compared with BVM, LT^®^evo achieved higher tidal volumes, fewer insufficient ventilations, and shorter no-flow times. (iii) The device was rated highly for ease of handling, placement stability, and overall performance, comparable to TI but with faster deployment.

### No-flow-time and CPR quality

The importance of no-flow-time as an essential prerequisite for survival and a favourable neurological outcome has been established over the past decades [10–12]. The association between minimized interruptions in chest compressions and improved survival has been consistently demonstrated in observational studies [13, 14]. The ERC guidelines recommend minimizing interruptions to chest compressions as much as possible, even when performing airway management [4]. While BVM remains a fundamental technique at the start of ACLS, it often requires pauses in compressions to achieve adequate ventilation. Studies have demonstrated that asynchronous BVM frequently results in suboptimal tidal volumes and prolonged hands-off intervals, which likely explains the longer no-flow time observed with BVM in this study [15, 16]. In contrast, the LT^®^evo can be inserted rapidly and allows ventilation to proceed with minimal interruption of chest compressions. However, differences in no-flow time during our simulation primarily reflect process-related aspects of airway management within a standardized resuscitation algorithm rather than isolated device performance. Also, the absolute difference observed between BVM and LT^®^evo was small (approximately 4 s) and should therefore be interpreted cautiously, as statistical significance does not necessarily imply clinical relevance in a controlled simulation setting.

### Airway management and provider competence

While TI is widely used as an advanced airway technique, its performance during CPR depends on provider competence and may be associated with increased interruption times, particularly in prehospital settings. Moreover, TI is known to be associated with significantly longer no-flow times than SGA devices [9].

In this study, no-flow-times for TI and LT^®^evo were comparable, reflecting the high airway proficiency of active anaesthesiologists. In less experienced hands, such as paramedics or mixed emergency teams, TI generally produces longer delays and lower first-pass success rates [17, 18]. In such contexts, SGA devices are recommended as the preferred airway strategy by the ERC 2025 guidelines. While previous guidelines did not make any differentiated statements about a specific SGA, the updated guidelines now specify the i-gel^®^ as the first-choice SGA device. Laryngeal tubes and other SGAs are recommended when the i-gel^®^ is not available. This preference is based on the i-gel^®^’s ease of insertion, non-inflatable cuff design, and its demonstrated high first-attempt success rates in both clinical and prehospital settings [4, 11]. The present results demonstrate that a modern second generation SGA such as the LT^®^evo can deliver effective ventilation without compromising compression continuity in a simulated setting. Comparative studies of current second generation SGAs in clinical settings are therefore mandatory for future guidelines recommendations.

### Performance of the LT^®^evo

The LT^®^evo´s performance likely results from its design refinements compared with the established LTS-D, including a widened ventilation lumen allowing subsequent fibreoptic intubation, modified cuff geometry for improved oropharyngeal sealing, and a thermoplastic preformed curvature facilitating insertion. These enhancements may explain the observed ventilation parameters and high user ratings (See Fig. 1) [10, 12].

At the time the ERC 2025 guidelines were developed, the LT^®^evo - officially introduced to the market in November 2025 - was not yet available, leading to a lack of studies at this time on its clinical and emergency use. Therefore it could not be considered during the evaluation process. Our findings suggest that this second generation laryngeal tube type SGA warrants further clinical evaluation for airway management during resuscitation. If clinical studies verify our findings - rapid placement when compared to TI, and seal characteristics expected of modern SGAs - it might potentially broaden the available options for guideline-compliant airway management.

### Clinical implications

Studies have shown that ventilation and oxygenation during resuscitation measures are often ineffective, which is associated with negative outcomes [2, 4]. Since lay rescuers often only perform chest compression when providing CPR, adequate ventilation is particularly important for reoxygenation and ventilation when the emergency teams arrive [4].

In resuscitation scenarios such as those simulated here, the use of BVM remains appropriate when effective ventilation - defined by the ERC guidelines as visible chest rise - is achievable. Although all participants were experienced anaesthesiologists, BVM exhibited greater variability in performance and a higher incidence of suboptimal ventilations. TI provided consistent ventilation but required more time until first ventilation via a secured airway. In contrast, the LT^®^evo was associated with short insertion times, fewer insufficient ventilations, and favourable user ratings within this controlled simulation setting.

### Limitations

This study has several important limitations. First, as a simulation-based investigation, it was conducted in a controlled environment without the physiological or psychological variables present in real cardiac arrests. The manikin used represented a standard, non-obstructed airway without secretions, regurgitation, aspiration or anatomical variation, therefore, the results may not fully translate to a clinical setting. Second, all participants were highly trained anaesthesiologists, which likely reduced variability in the airway performance and may not reflect outcomes or performance among less experienced providers such as paramedics or emergency physicians in training. Furthermore, delivered tidal volumes may not exclusively reflect device-specific performance but could also be influenced by individual ventilation technique, despite standardized instructions and statistical adjustment. Third, the sample size was relatively small (*n* = 20). Finally, the study did not include clinical endpoints such as return of spontaneous circulation or survival, which limits inference to procedural and performance parameters.

Nevertheless, simulation studies remain a validated method for evaluating new airway devices, particularly when ethical or logistical barriers prevent immediate clinical trials. The controlled setting allowed for standardized comparison of airway techniques and precise measurement of no-flow time, offering an essential foundation for subsequent patient-based investigations.

Finally, manufacturer involvement should be acknowledged. Although the sponsor had no influence on study design, data analysis, or interpretation, the results of this simulation-based feasibility study should be interpreted within their methodological context.

## Conclusion

In this randomised, crossover manikin study, the LT^®^evo enabled rapid, effective airway management and ventilation with minimal interruption of chest compressions compared with BVM and TI. Within this simulated scenario, the device achieved, the highest ventilation quality, and received high ratings for handling and stability by experienced anaesthesiologists. The fact that it could be used correctly and effectively without extensive prior training could make it a promising tool for users in the medical emergency field who are less experienced in airway management.

These findings suggest that the LT^®^evo represents a promising second-generation SGA device for resuscitation scenarios. Further clinical and prehospital studies are warranted to confirm its effectiveness and safety under real-world conditions.

## Data Availability

The datasets generated and analysed during the current study are not publicly available due to institutional data protection regulations but are available from the corresponding author (MV) on reasonable request.

## Abbreviations

ACLS: Advanced Cardiac Life Support
ALS: Advanced Life Support
BVM: Bag-Valve-Mask Ventilation
CI: Confidence Interval
CPR: Cardiopulmonary Resuscitation
ERC: European Resuscitation Council
EMS: Emergency Medical Service
IQR: Interquartile Range
LT: Laryngeal Tube
LT®evo: Laryngeal Tube Evo (VBM Medizintechnik GmbH, Germany)
LTS-D: Laryngeal Tube Suction Disposable
OHCA: Out-of-Hospital Cardiac Arrest
RC: Regression Coefficient
SGA: Supraglottic Airway
TI: Tracheal Intubation
